# Early serodiagnosis of trichinellosis by ELISA using excretory–secretory antigens of *Trichinella spiralis* adult worms

**DOI:** 10.1186/s13071-015-1094-9

**Published:** 2015-09-23

**Authors:** Ge-Ge Sun, Zhong-Quan Wang, Chun-Ying Liu, Peng Jiang, Ruo-Dan Liu, Hui Wen, Xin Qi, Li Wang, Jing Cui

**Affiliations:** Department of Parasitology, Medical College, Zhengzhou University, Zhengzhou, China

**Keywords:** *Trichinella spiralis*, Trichinellosis, Adult worm, Excretory–secretory (ES) antigens, Serodiagnosis

## Abstract

**Background:**

The excretory–secretory (ES) antigens of *Trichinella spiralis* muscle larvae (ML) are the most commonly used diagnostic antigens for trichinellosis. Their main disadvantage for the detection of anti-*Trichinella* IgG is false-negative results during the early stage of infection. Additionally, there is an obvious window between clinical symptoms and positive serology.

**Methods:**

ELISA with adult worm (AW) ES antigens was used to detect anti-*Trichinella* IgG in the sera of experimentally infected mice and patients with trichinellosis. The sensitivity and specificity were compared with ELISAs with AW crude antigens and ML ES antigens.

**Results:**

In mice infected with 100 ML, anti-*Trichinella* IgG were first detected by ELISA with the AW ES antigens, crude antigens and ML ES antigens 8, 12 and 12 days post-infection (dpi), respectively. In mice infected with 500 ML, specific antibodies were first detected by ELISA with the three antigen preparations at 10, 8 and 10 dpi, respectively. The sensitivity of the ELISA with the three antigen preparations for the detection of sera from patients with trichinellosis at 35 dpi was 100 %. However, when the patients’ sera were collected at 19 dpi, the sensitivities of the ELISAs with the three antigen preparations were 100 % (20/20), 100 % (20/20) and 75 % (15/20), respectively (*P* < 0.05). The specificities of the ELISAs with the three antigen preparations were 98.11, 95.60 and 89.31 %, respectively (*P* < 0.05).

**Conclusions:**

The sensitivity and specificity of the *T. spiralis* AW ES antigens were superior to those of the AW crude antigens and ML ES antigens. Thus, the AW ES antigens might serve as potential antigens for the early and specific serodiagnosis of trichinellosis.

## Background

Trichinellosis is a serious zoonotic disease caused by the nematode genus *Trichinella*. Humans acquire the disease by ingesting raw or insufficiently cooked meat of pigs or other animals containing the *Trichinella* larvae [1]. Human trichinellosis has been documented in 55 countries around the world and is considered to be a neglected tropical disease, especially in developing countries [2, 3]. From 2004 to 2009, 15 outbreaks of human trichinellosis consisting of 1387 cases and 4 deaths were reported in China [4]. Trichinellosis is a major food-borne zoonosis with health, social, and economic impacts in endemic countries [5].

Following the ingestion of infested meat, *Trichinella* larvae are released from their capsules to invade the upper small intestine and mature into adult worms (AW). The hallmark of the intestinal phase, which usually lasts 1 week, is a non-specific gastroenteritis, with diarrhoea, abdominal pain, nausea, and/or vomiting. In 2–3 weeks, the fertilized females produce ~1500 newborn larvae that migrate via the blood and lymphatic systems to invade and encapsulate in the skeletal muscles (muscular or acute phase). The acute phase is associated with an inflammatory and allergic response to muscle invasion by the migrating larvae. Fever, eyelid or facial oedema, myalgia, and eosinophilia are the most prominent manifestations [6, 7].

However, the clinical diagnosis of human trichinellosis is difficult because its clinical manifestations are nonspecific [6, 7]. At present, a definitive diagnosis of human trichinellosis can be made by detecting larvae in a muscle biopsy sample or specific anti-*Trichinella* IgG antibodies [8], but the parasitological examination of biopsy samples is not sufficiently sensitive to detect *Trichinella* larvae in light infections and during the early stage of infection. Because *T. spiralis* muscle larvae (ML) are easily collected from experimentally infected animals, their excretory–secretory (ES) antigens can be prepared by the *in vitro* cultivation of isolated muscle larvae, resulting in a preparation with high specificity [9]. Thus, an ELISA with the ML ES antigens is the most commonly used serological method for the diagnosis of trichinellosis and is recommended by the International Commission on Trichinellosis (ICT) [10, 11]. The main disadvantage of the detection of anti-*Trichinella* IgG against ML ES antigens is the occurrence of a high rate of false negative results during the early stage of infection [12]. These false negatives may occur because the majority of *T. spiralis* ML ES antigens are stage-specific; thus, the ML ES antigens are not recognized by antibodies induced by the parasites during the intestinal phase [13, 14]. Several studies demonstrated that the maximum detection rate of 100 % of anti-*Trichinella* IgG was not reached until at least 1–3 months after human infection with the parasite [10]. There is an obvious time lag (window period) between the time when patients develop clinical symptoms and positive serology during the acute stage of trichinellosis [6, 15]. Additionally, the ML ES antigens also have cross-reactivity with the sera of patients with other helminthiases (e.g., paragonimiasis, schistosomiasis, clornorchiasis, cysticercosis, and anisakiasis) [15–17]. Hence, there is an urgent need to develop new specific early diagnostic antigens for acute trichinellosis.

*Trichinella spiralis* ML develop to AW in the small intestine 31 h after infection, live in the intestinal mucosa and persist for 10–20 days in mice and rats or 4–6 weeks in humans [18]. During the intestinal stage of trichinellosis, the ES antigens produced by the AW result in early exposure to the immune system and elicit the production of specific anti-*Trichinella* antibodies by the host. Previous studies showed that AW crude antigens were recognized by infected sera from mice or swine 7 days post-infection (dpi) [19]. However, to the best of our knowledge there has been no report on the serodiagnosis of trichinellosis using *T. spiralis* AW ES antigens. The aim of this study was to evaluate the potential of *T. spiralis* AW ES antigens for the early serodiagnosis of acute trichinellosis.

## Methods

### Ethics statement

All procedures of animal experiments and the use of the patients’ serum samples in this study were approved by the Life Science Ethics Committee of Zhengzhou University (no. 2011–016). Before the investigation, oral informed consent was obtained from all individuals.

### Parasites and experimental animals

The isolate (ISS534) of *T. spiralis* used in this study was obtained from domestic pigs in Nanyang, Henan Province, China. The reference *Trichinella* isolates used in this study were *T. nativa* (T2, ISS10), *T. britovi* (T3, ISS100), *T. pseudospiralis* (T4, ISS13) and *T. nelsoni* (T7, ISS29); these isolates were obtained from the International *Trichinella* Reference Centre (ITRC; Rome, Italy). The *Trichinella* isolates were maintained by serial passage in BALB/c mice every 6–8 months in our laboratory. Specific pathogen-free (SPF) female BALB/c mice aged 6 weeks were purchased from the Experimental Animal Centre of Henan Province.

### Serum samples

*T. spiralis* was used to infect the BALB/c mice. Twenty mice were randomly divided into two groups (ten mice per group): mice infected with 500 ML and mice infected with 100 ML. Approximately 100 μl of tail blood was collected from the infected mice on alternate days from 2–28 dpi to isolate sera [12]. Serum samples from normal mice were used as the negative control.

Sera were obtained from BALB/c mice infected with 300 ML of *T. spiralis*, *T. nativa*, *T. britovi*, *T. pseudospiralis* and *T. nelsoni* 42 dpi. Serum samples of mice infected with three spargana of *Spirometra mansoni* were collected 30 dpi in our department. Serum samples of mice infected with *Toxoplasma gondii, Schistosoma japanicum* and *Angiostrongylus cantonensis* were gifted by Prof. GR Yin (Shanxi Medical University), Dr. JH Lei (Tongji Medical College of Huazhong University of Science and Technology), and Prof. ZY Lv (Department of Parasitology, Zhongshan School of Medicine, Sun Yat-Sen University), respectively. All of the serum samples were stored at −80 °C prior to use.

Forty-two serum samples from patients with trichinellosis were used to identify the sensitivity of the *T. spiralis* AW ES antigens*.* These serum samples were collected from the patients during two outbreaks with trichinellosis that occurred in the Yunnan province of southwestern China in 2003 and 2013 [20, 21]. These patients with trichinellosis presented with a clinical suspicion of trichinellosis. The diagnosis of trichinellosis was confirmed for all of these patients by the presence of a high fever associated with periorbital or facial oedema, myalgia, a high level of eosinophilia, and a history of ingestion of raw or undercooked meat. All of these patients had positive ELISA or IIF results for trichinellosis, and two patients had a *Trichinella*-positive muscle biopsy. Out of 42 patients with trichinellosis, 22 serum samples were collected 35 dpi and 20 were collected 19 dpi.

Sera used to assess cross-reactivity were selected from 109 patients with other parasitic diseases confirmed by parasitological examination of faeces, histopathological examination of biopsy samples or positive specific serological test results: schistosomiasis (36 patients with *Schistosoma japonicum* infection), paragonimiasis (20 patients with *Paragonimus skrjabini* infection), clonorchiasis (7 patients with *Clonorchis sinensis*), cystic echinococcosis (21 patients), *Taenia solium* cysticercosis (18 patients), and *Spirometra erinaceieuropaei* sparganosis (7 patients). These patients were diagnosed and serum samples were collected in our department [22, 23]. Serum samples from 50 presumably healthy persons who tested negative for the above-mentioned parasitic diseases were also included in the study. All of the serum samples were stored at −80 °C prior to use.

### Collection of worms and preparation of ES antigens

One hundred mice were orally infected with 5000 ML of *T. spiralis* and euthanized 3 dpi. The small intestine was collected, cut along its entire length, and washed in pre-warmed PBS. Then, the small intestine was cut into pieces and incubated in PBS at 37 °C for 1.5 h on a 300 μm sieve. The released AW were separated from the intestinal debris by filtration through a 200 μm sieve and differential sedimentation for 30 min. After several washes in PBS supplemented with 100 U penicillin/ml and 100 μg streptomycin/ml, the worms were centrifuged at 600 × g for 10 min and collected [19, 24, 25]. The average number of AW recovered from the infected mice was approximately 1000 worms per mouse.

AW ES antigens were prepared as previously described [9, 26]. Briefly, after thorough washing in sterile saline and serum-free RPMI-1640 medium supplemented with 100 U penicillin/ml and 100 μg streptomycin/ml, the AW were incubated in the same medium at a density of 2000 worms/ml at 37 °C in 5 % CO_2_ for 18 h. After incubation, the media containing the ES proteins were poured into 50-ml conical tubes, and the worms were allowed to settle for 20 min. The supernatant containing the ES products was filtered through a 0.2 μm membrane. The ES products were dialyzed and then lyophilized by vacuum concentration and freeze-drying (Heto-Holten A/S, Denmark). The protein concentration of the ES antigens of the adult worms was determined by the Bradford assay [27].

*T. spiralis* ML were recovered from the infected mice 42 dpi by artificial digestion [28, 29]. The ML ES antigens were prepared as described above.

The crude (somatic) antigens of *T. spiralis* AW were prepared as previously described [30, 31]. Briefly, adults were resuspended in deionized water. The suspension was subjected to 5 freeze-thaw cycles. The worms were homogenized on ice in a glass tissue grinder. Then, the larval fragments were further homogenized with ultrasonication (99 3-s cycles, 100 W, 0 °C). The supernatant was collected after centrifugation at 15,000 × g for 1 h at 4 °C. The protein concentration of the AW crude antigens was determined by the Bradford assay [27].

### Enzyme-linked immunosorbent assay

The optional dilutions of various reagents were determined using checkerboard titration. ELISA was performed as previously described [15, 32]. Briefly, 96-well ELISA plates (Corning, USA) were coated with AW ES antigens (2.0 μg/ml), ML ES antigens (2.5 μg/ml) or AW crude antigens (1.5 μg/ml) in 100 μl of bicarbonate buffer (pH 9.6) overnight at 4 °C. After blocking with PBS-0.1 % Tween 20 (PBST) containing 5 % skimmed milk at 37 °C for 2 h, the following reagents were sequentially added and incubated at 37 °C for 1 h: (1) human or mouse sera diluted 1:200 or 1:100, respectively, in PBST, and (2) HRP-conjugated anti-human or anti-mouse IgG (Sigma, USA) diluted 1:5000. The reactions were detected by the addition of the substrate o-phenylenediamine dihydrochloride (OPD; Sigma, USA) plus H_2_O_2_ and stopped with 50 μl/well of 2 M H_2_SO_4_. Optical density (OD) values at 490 nm were measured with a microplate reader (TECAN, Austria). All samples were run in duplicate.

The cut-off value of the ELISA was evaluated for the three antigen preparations based on a 2.1-fold increase over the average OD value of the negative samples. Ratios <2.1 of the samples to be tested/negative sample (OD values of the samples to be tested divided by OD of the negative, S/N < 2.1) were regarded as negative, whereas S/N ≥2.1 was regarded as positive [15, 23, 33]. A test was considered valid when: (1) the OD values of the negative controls were higher than the OD values of the blank controls; and (2) the OD values of the negative controls were lower than the cut-off values. The sensitivity and specificity of the ELISA were assessed according to the following formulas: Sensitivity = no. of true positives/(no. of true positives + no. of false negatives) × 100 and Specificity = no. of true negatives/(no. of false positives + no. of true negatives) × 100 [34].

### Statistical analysis

All statistical analyses were performed with SPSS for Windows, version 17.0 (SPSS Inc., Chicago, IL, USA). The sensitivity and specificity of the AW ES, crude antigens and ML ES antigens were compared with the Chi-square test. Repeated-measures analysis of variance was used to determine the difference between antibody levels at various time points post-infection and with different infecting doses. *P* < 0.05 was considered statistically significant.

## Results

### The ELISA cut-off values

The cut-off values of the ELISA with the AW ES antigens, crude antigens and ML ES antigens for the detection of experimentally infected mice were 0.19, 0.23 and 0.20, respectively. The cut-off values of the ELISA with the above three antigen preparations for the detection of patients with trichinellosis were 0.41, 0.47 and 0.45, respectively.

### Detection of anti-*Trichinella* IgG in mice infected with *Trichinella* and other parasites

The levels of anti-*Trichinella* IgG in the serum samples of mice infected with *T. spiralis* and other parasites were determined; the results are shown in Table 1. The sensitivity of the ELISA with the AW ES antigens, crude antigens and ML ES antigens for the detection of anti-*Trichinella* IgG in the sera of mice infected with 300 *T. spiralis* ML collected at 42 dpi was 100.00 % (35/35). No cross-reactivity of the ELISAs with the three antigen preparations was observed with the sera of mice infected with *Angiostrongylus cantonensis*, *Spirometra mansoni*, *Schistosoma japonicum*, and *T. gondii* and normal mice.

**Table 1:** Detection of serum anti-*Trichinella* IgG in mice infected with *Trichinella spiralis* and other parasites by ELISA with the three *T. spiralis* antigen preparations

Sera of mice infected with	No. of serum samples	ELISA with AW ES antigens		ELISA with AW crude antigens		ELISA with ML ES antigens	
		OD value $\left(\overline{\mathrm{X}}\pm S\right)$	No. of positive serum samples (%)	OD value $\left(\overline{\mathrm{X}}\pm S\right)$	No. of positive serum samples (%)	OD value $\left(\overline{\mathrm{X}}\pm S\right)$	No. of positive serum samples (%)
*T. spiralis*	35	0.60 ± 0.11	35 (100)	0.43 ± 0.09	35 (100)	0.58 ± 0.13	35 (100)
*A. cantonensis*	12	0.06 ± 0.01	0 (0)	0.05 ± 0.01	0 (0)	0.03 ± 0.01	0 (0)
*S. mansoni*	26	0.11 ± 0.04	0 (0)	0.12 ± 0.04	0 (0)	0.15 ± 0.03	0 (0)
*S. japonicum*	16	0.15 ± 0.05	0 (0)	0.12 ± 0.03	0 (0)	0.15 ± 0.06	0 (0)
*T. gondii*	6	0.10 ± 0.04	0 (0)	0.11 ± 0.05	0 (0)	0.14 ± 0.01	0 (0)
Normal mice	46	0.09 ± 0.02	0 (0)	0.12 ± 0.02	0 (0)	0.09 ± 0.02	0 (0)

The levels of anti-*Trichinella* IgG in mice infected with other *Trichinella* species were assessed by ELISA; the results are shown in Table 2. The detection rates of anti-*Trichinella* IgG by ELISA with the AW and ML ES antigens were 100 % in mice infected with *T. nativa*, *T. britovi* and *T. nelsoni*. However, the antibody detection rate (90.91 %) of mice infected with *T. pseudospiralis* by ELISA with the ML ES antigens was 9.09 % higher than the ELISA with the AW ES antigens (*χ*^*2*^ = 29.455, *P* < 0.05). When the sera of mice infected with *T. nativa*, *T. britovi*, *T. nelson* or *T. pseudospiralis* were assessed by ELISA with the AW crude antigens, the antibody detection rates were significantly lower compared to the ELISA with the ML ES antigens (*χ*^*2*^_T2_ = 29.939, *χ*^*2*^_T3_ = 17.368, *χ*^*2*^_T4_ = 36.667, and χ^2^_T7_ = 14.545, respectively, *P* < 0.05.

**Table 2:** Detection of serum anti-*Trichinella* IgG in mice infected with other *Trichinella* species by ELISA with the three *T. spiralis* antigen preparations

Sera of mice infected with	No. of serum samples	ELISA with AW ES antigens		ELISA with AW crude antigens		ELISA with ML ES antigens	
		OD value $\left(\overline{\mathrm{X}}\pm S\right)$	No. of positive serum samples (%)	OD value $\left(\overline{\mathrm{X}}\pm S\right)$	No. of positive serum samples (%)	OD value $\left(\overline{\mathrm{X}}\pm S\right)$	No. of positive serum samples (%)
*T. nativa*	26	0.34 ± 0.12	26 (100)	0.18 ± 0.08	7 (26.92)	0.71 ± 0.13	26 (100)
*T. britovi*	15	0.40 ± 0.12	15 (100)	0.20 ± 0.12	4 (26.67)	0.79 ± 0.08	15 (100)
*T. pseudospiralis*	22	0.06 ± 0.07	2 (9.09)	0.06 ± 0.02	0 (0)	0.52 ± 0.21	20 (90.91)
*T. nelsoni*	16	0.31 ± 0.09	16 (100)	0.20 ± 0.14	6 (37.50)	0.76 ± 0.08	16 (100)

### Serum anti-*Trichinella* IgG dynamics in mice experimentally infected with different numbers of parasites

**Fig. 1:**
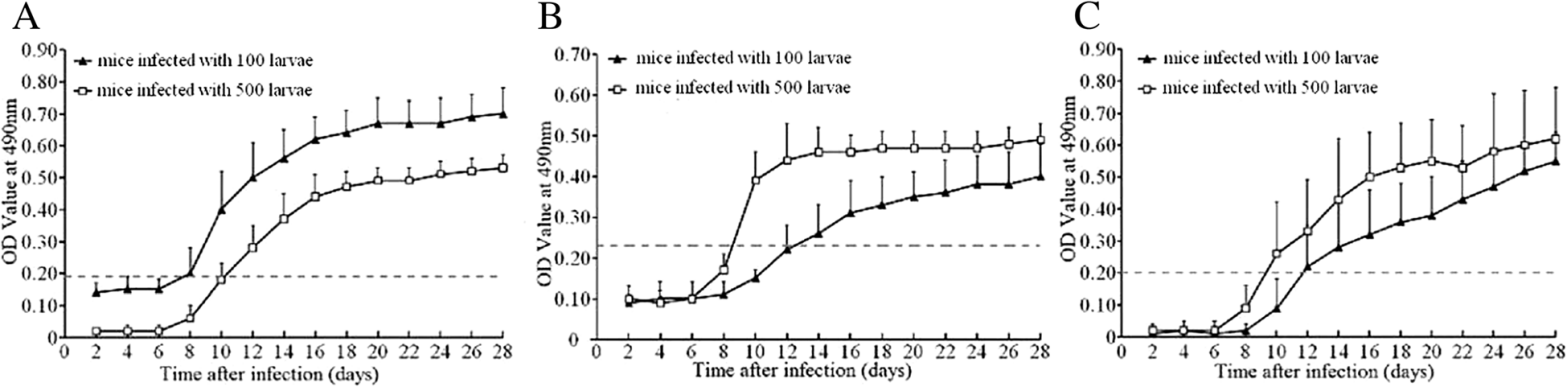
Kinetics of anti-*Trichinella* IgG in the sera of mice infected with 500 and 100 muscle larvae. Anti-*Trichinella* IgG was detected by ELISA with adult worm ES antigens (**a**), adult worm crude antigens (**b**) and muscle larval ES antigens (**c**). The cut-off value is represented by the dotted line

**Fig. 2:**
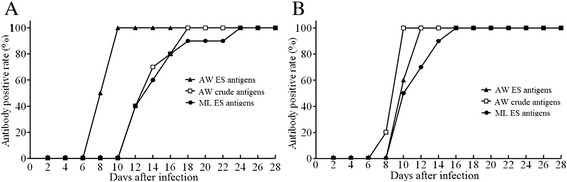
Comparison of the detection rate of anti-*Trichinella* IgG in the sera of mice infected with different numbers of larvae by ELISA with the three antigen preparations. **a** mice infected with 100 larvae. **b** mice infected with 500 larvae

**Fig. 3:**
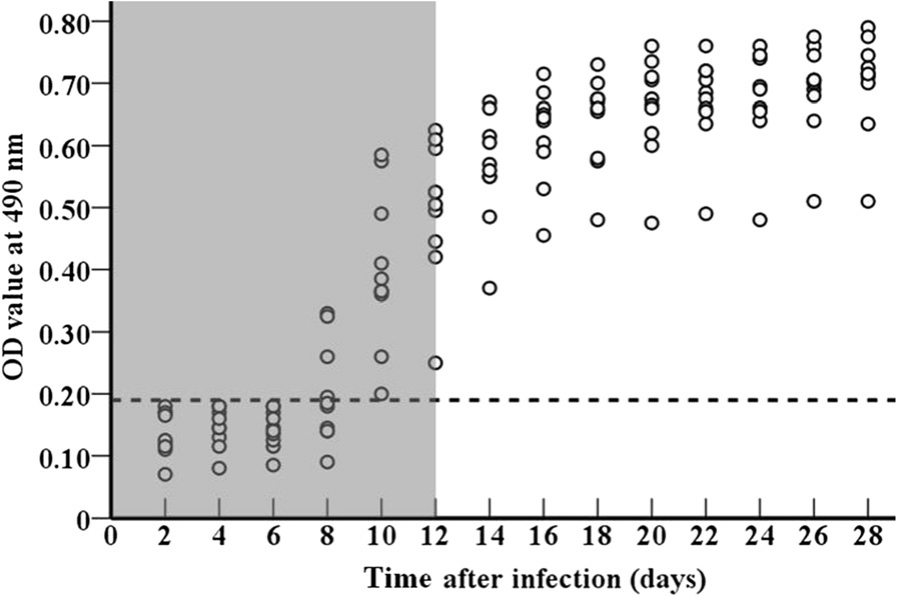
Optical density values from the ELISA with adult worm ES antigens of mice infected with 100 *T. spiralis* muscle larvae. The cut-off value is represented by the dotted line. The window where the detection of anti-*Trichinella* IgG is not possible by ELISA with the muscle larval ES antigens is presented in grey

The levels of anti-*Trichinella* IgG in the sera from the 2 groups of infected mice at different time points after infection were determined by ELISA with the three antigens preparations (Fig. 1). The results showed that the serum anti-*Trichinella* IgG levels in mice infected with 100 and 500 larvae began to increase rapidly 8 dpi and continued to elevate throughout the experimental period. There were significant differences in the anti-*Trichinella* IgG levels at different time points post-infection in the 2 groups of infected mice (*F*_WA ES_ = 408.307, F_AW crude_ = 204.921, and F_ML ES_ = 165.990, *P* < 0.05). When the AW ES antigens were used, the anti-*Trichinella* IgG levels in the mice infected with 100 larvae were higher than the mice infected with 500 larvae (*F* = 68.023, *P* < 0.05). However, when the AW crude antigens and ML ES antigens were used, the anti-*Trichinella* IgG levels in the mice infected with 500 larvae were obviously higher compared to the mice infected with 100 larvae (F_AW crude_ = 38.118 and F_ML ES_ = 5.999*, P* < 0.05). In the mice infected with 100 ML, anti-*Trichinella* IgG was first detected by ELISA with the AW ES antigens, crude antigens and ML ES antigens 8, 12 and 12 dpi with detection rates of 50, 40 and 40 %, respectively; the antibody positivity rates reached 100 % on 10, 18 and 24 dpi, respectively (Figs. 2a and 3). In the mice infected with 500 ML, anti-*Trichinella* IgG was first detected by ELISA with the three antigens on 10, 8 and 10 dpi with detection rates of 60, 20 and 50 %, respectively; the antibody positivity rates reached 100 % on 12, 10 and 16 dpi, respectively (Fig. 2b).

### Detection of anti-*Trichinella* IgG in serum samples of patients with trichinellosis and other parasitoses

The sensitivity of ELISA with the AW ES antigens, crude antigens and ML ES antigens for the detection of anti-*Trichinella* IgG in the sera of patients with trichinellosis was 100 % on 35 dpi (22/22) (Table 3). However, when the patients’ sera were collected 19 dpi, the sensitivity of the ELISAs with the three antigens was 100 % (20/20), 100 % (20/20) and 75 % (15/20), respectively (χ^2^ = 10.909, *P* < 0.05). The specificity of the ELISAs with the three antigens was 98.11 % (156/159), 95.60 % (152/159) and 89.31 % (142/159), respectively (χ^2^ = 12.249, *P* < 0.05), when performed with the sera of patients with other parasitic diseases and healthy persons. No cross-reactions of the ELISA with the AW ES antigens and crude antigens were observed with the sera of patients with schistosomiasis, clonorchiosis, echinococcosis, and sparganosis, and healthy persons. However, the cross-reactivity (25 %) of the ELISA with the AW crude antigens with the sera of patients with paragonimiasis was higher than 10 % with the AW ES antigens, although the difference was not statistically significant (χ^2^ = 1.558, *P >* 0.05).

**Table 3:** Detection of anti-*Trichinella* IgG in serum samples of patients with trichinellosis and other parasitoses by ELISA with the three *T. spiralis* antigen preparations

Sera of patients with	No. of serum samples	ELISA with AW ES antigens		ELISA with AW soluble antigens		ELISA with ML ES antigens	
		OD value $\left(\overline{\mathrm{X}}\pm S\right)$	No. of positive serum samples (%)	OD value $\left(\overline{\mathrm{X}}\pm S\right)$	No. of positive Serum samples (%)	OD value $\left(\overline{\mathrm{X}}\pm S\right)$	No. of positive serum samples (%)
Later trichinellosis^a^	22	0.52 ± 0.10	22 (100)	0.54 ± 0.05	22 (100)	0.68 ± 0.04	22 (100)
Early trichinellosis^b^	20	0.56 ± 0.12	20 (100)	0.62 ± 0.08	20 (100)	0.53 ± 0.10	15 (75.00)
Schistosomiasis	36	0.25 ± 0.06	0 (0)	0.30 ± 0.07	0 (0)	0.33 ± 0.10	4 (20.00)
Paragonimiosis	20	0.29 ± 0.11	2 (10.00)	0.40 ± 0.14	5 (25.00)	0.26 ± 0.14	1 (5.00)
Clonorchiosis	7	0.21 ± 0.04	0 (0)	0.22 ± 0.05	0 (0)	0.30 ± 0.11	1 (14.29)
Cysticercosis	18	0.28 ± 0.10	1 (5.56)	0.29 ± 0.12	2 (11.00)	0.38 ± 0.12	5 (25.00)
Echinococcosis	21	0.27 ± 0.07	0 (0)	0.28 ± 0.07	0 (0)	0.35 ± 0.14	5 (25.00)
Sparganosis	7	0.26 ± 0.04	0 (0)	0.34 ± 0. 04	0 (0)	0.27 ± 0.10	1 (12.5)
Healthy persons	50	0.19 ± 0.03	0 (0)	0.23 ± 0.04	0 (0)	0.21 ± 0.08	0 (0)

Later trichinellosis^a^: The sera of later patients with trichinellosis were collected 35 days post-infection

Early trichinellosis^b^: The sera of patients with early-stage trichinellosis were collected 19 days post-infection

## Discussion

In the present study, the AW ES antigens of *T. spiralis* were used as diagnostic antigens for the detection of anti-*Trichinella* IgG in the sera of experimentally infected mice. The sensitivity and specificity of the ELISA with the AW ES antigens were 100 % (35/35) and 100 % (106/106) in the infected mice, respectively. Moreover, there was no significant difference in the antibody detection rates in the sera of mice infected with *T. nativa*, *T. britovi*, and *T. nelsoni* by ELISA with the AW ES antigens and ML ES antigens (*P* > 0.05). The results suggested that there were common AW ES antigens among the encapsulated *Trichinella* species (i.e., *T. spiralis*, *T. nativa*, *T. britovi*, and *T. nelsoni*), but not in the non-encapsulated *Trichinella* species (*T. pseudospiralis*) [35, 36]. Moreover, most of the epitopes of the AW ES antigens recognized by sera from infected mice were common to the encapsulated *Trichinella* species [37]. Our results indicated that the AW ES antigens of *T. spiralis* could also be used for the serodiagnosis of trichinellosis caused by other encapsulated *Trichinella* species.

Our ELISA results showed that the antigenic epitopes of the AW ES antigens were recognized by sera from infected mice 8–12 dpi, suggesting that the AW ES antigens might be secreted into the peripheral blood circulation of the host during the intestinal stage of *Trichinella* infection and induce an early antibody response that continues into the muscle larval stage [15]. Importantly, the anti-*Trichinella* IgG in 50 % of the mice infected with 100 larvae was detected by ELISA with the AW ES antigens as soon as 8 dpi, whereas the ELISA with the ML ES antigens did not permit detection of the infected mice before 12 dpi. In contrast, anti-*Trichinella* IgG in the mice infected with 500 larvae was first detected by ELISA with the AW ES antigens on 10 dpi. The mechanism underlying the earlier detection of anti-*Trichinella* IgG by the AW ES antigens in mice infected with 100 larvae compared to mice infected with 500 larvae is unclear, but may be due to the suppression of the host’s immune response to the AW ES antigens by the higher infecting doses during the early stage of *Trichinella* infection [38, 39]. Other studies showed that the recombinant proteins from the early adult worms 20 h post-infection were recognized by Western blotting as soon as 15–20 dpi in sera from pigs experimentally infected with 20,000 *T. spiralis* muscle larvae [40]. These results suggested that the AW ES antigens were more sensitive than the recombinant AW antigens for the serodiagnosis of trichinellosis. Thus, the AW ES antigens of *T. spiralis* exhibit potential for the early diagnosis of trichinellosis.

The sensitivity of the ELISAs with the AW ES antigens and ML ES antigens towards early patient serum samples collected on 19 dpi were 100 and 75 % (*P* < 0.05), respectively, suggesting that the AW ES antigens had early serodiagnostic value for human trichinellosis.

Cross-reactive antigens from different species of helminths have been known since the initial studies of Capron et al*.* [41]. Previous studies showed that the ELISA with the ML crude antigens exhibited obvious cross-reactivity with the sera of patients infected with other helminths [42]. Although the use of ML ES antigens could increase the sensitivity of the ELISA, 39.2 % of the false-positive results were observed with sera from patients with other parasitic diseases (cysticercosis, hydatidosis, schistosomiasis, fascioliasis, strongyloidosis, toxocariasis, anisakiasis or filariasis) [17]. When synthetic tyvelose antigens of *T. spiralis* ML were used for the serodiagnosis of trichinellosis, cross-reactivity occurred with cases of anisakiasis [16]. Recently, recombinant antigens of *T. spiralis* ML have been developed in some laboratories; however, the recombinant Ts21 or Ts 43 antigens still had a high occurrence of cross-reactivity with the sera of patients with schistosomiasis, paragonimiasis, clonorchiasis, echinococcosis or cysticercosis [43, 44].

Western blotting of crude or ES antigens of *T. spiralis* ML is the serological method for the confirmatory test for trichinellosis recommended by the ICT. When specific IgG antibodies against the ML antigen components (40–70 kDa) are detected, the diagnosis of *Trichinella* infection is confirmed [10]. Western blotting with the ML crude or ES antigens is mainly used to confirm ELISA-positive sera from animals and humans. The sensitivity and specificity of this technique range from 96 to 99 % and from 95.6 to 99.6 %, respectively [45, 46]. Hence, Western blotting has been widely applied to the serodiagnosis and seroepidemiological investigation of *Trichinella* infection in humans and animals [47–50]. However, the 40–70 kDa protein bands of the *T. spiralis* ML also exhibited cross-reactivity with the sera of patients with other helminthiases [17, 51]. Moreover, shared antigens have been identified for *T. spiralis* ML and several other parasitic worms (*Trichuris* spp., *Fasciola hepatica, Schistosoma mansoni, Schistosoma japonicum, Paragonimus westermani* and *Clornorcis sinensis*) [52–55]. Anti-gp50 monoclonal antibodies against schistosomes and anti-gp50-positive sera from patients with schistosomiasis cross-reacted with the hypodermis and stichocytes of the *T. spiralis* ML [56]. These results demonstrated that different kinds of antigens from the *T. spiralis* ML had partial cross-reactivity with the sera of patients with other helminthiases. Hence, new sources of diagnostic antigens from other stages of *T. spiralis* should be developed to improve the sensitivity and specificity of the serodiagnosis of trichinellosis.

Our results showed that the specificity of the *T. spiralis* AW ES antigens was significantly higher than the ML ES antigens. No cross-reactions of the AW ES antigens were observed with the sera of patients with schistosomiasis, clonorchiosis, echinococcosis, or sparganosis or healthy persons. The AW ES antigens were sensitive and specific for the detection of anti-*Trichinella* IgG in serum samples of patients in the early stage of trichinellosis. The application of AW ES antigens for the serodiagnosis of trichinellosis could remarkably shorten the window when the detection of anti-*Trichinella* IgG is not possible by the ELISA with ML ES antigens. The AW ES antigens are a good alternative to the ML ES antigens and should be considered as a potential specific diagnostic antigen preparation for early trichinellosis.

However, cross-reactivity of the ELISA with the AW ES antigens was observed with 5.56 % (1/18) of patients with cysticercosis and 10 % (2/20) of patients with paragonimiosis. Hence, a large-scale validation of the specificity of the AW ES antigens needs to be performed with the sera of the patients with other helminthiases. Moreover, the *Trichinella-*specific protein bands in the AW ES antigens should be identified and validated by Western blotting, and specific recombinant AW antigens should be developed in the future.

## Conclusions

This study demonstrated that the AW ES antigens of *T. spiralis* were sensitive and specific for the early diagnosis of trichinellosis. These antigens provided a new source of diagnostic antigens and might serve as potential antigens for the early and specific serodiagnosis of trichinellosis. However, these results need to be further evaluated with a large-scale trial using the sera of patients with trichinellosis and other helminthiases.
